# TEKIBEN --- DIGITAL DISIMPACTION AT HOME CARE IN JAPAN

**DOI:** 10.1093/geroni/igad104.3622

**Published:** 2023-12-21

**Authors:** Shoko Miyagawa, Young Ah Seong

**Affiliations:** Keio University, Fujisawa, Kanagawa, Japan; Hosei University, Tokyo, Japan

## Abstract

Though this may not be the case worldwide, in Japan, digital disimpaction (Tekiben in Japanese) is one of the most frequently performed care procedures done by home-visit nurses. This study aimed to clarify the status of digital disimpaction at home care in Japan by interviewing nurses skilled in disimpaction. The interviews were conducted during the process of developing a simulator for digital disimpaction training and were conducted with seven nurses who provide home care. The interviews lasted between 50 and 75 minutes each. The interview data were transcribed, and descriptions of the frequency and target audience for digital disimpaction were extracted. The frequency with which each nurse performed digital disimpaction ranges from 10 to 30 times per month. The clients who require digital disimpaction include those who require the procedure regularly due to conditions such as cerebral infarction or neuropathy, as well those who are in the terminal stages of cancer or other conditions and require the procedure when assessed as necessary. Digital disimpaction techniques are not taught in educational courses at universities or vocational schools but are learned through on-the-job training at hospitals or in-home nursing. The reason for performing routine digital disimpaction is that it is difficult for family members to assist with defecation. The importance of passing stool at the time of digital disimpaction is pointed out, as it is difficult for family members to handle bowel movements in the absence of a nurse.

